# The perception of the position of an unseen limb: Investigation of the effect of thixotropic conditioning on drift and accuracy

**DOI:** 10.1113/EP092686

**Published:** 2025-06-10

**Authors:** Simon C. Gandevia, Georgia Fisher, Joanna Diong, Annie A. Butler, Martin E. Héroux

**Affiliations:** ^1^ Neuroscience Research Australia Randwick New South Wales Australia; ^2^ University of New South Wales Sydney New South Wales Australia; ^3^ Centre for Healthcare Resilience and Implementation Science, Australian Institute of Health Innovation Macquarie University Sydney New South Wales Australia; ^4^ School of Medical Sciences, Faculty of Medicine and Health The University of Sydney Sydney New South Wales Australia

**Keywords:** healthy adults, perception, proprioception, proprioceptive drift, thixotropy

## Abstract

Proprioceptive judgements can be divided into two broad categories: low‐level and high‐level. Low‐level judgements of limb position require a person to detect, discriminate or match the position of a body part, whereas high‐level judgements require a person to report the position of an unseen body part relative to the external world. It has been suggested that muscle thixotropy – the influence of recent contraction or stretch on the passive properties of a muscle – impacts both the accuracy of low‐level judgements of limb position and the degree to which these judgements drift over time. However, high‐level proprioceptive judgements of upper limb position and the degree to which they drift over time may not be affected by thixotropy. This was investigated here. Twenty‐five healthy adult participants made visual judgements about the perceived position of their hidden index finger after their elbow muscles had been conditioned with a flexion or extension contraction, or after a series of large passive elbow movements. After conditioning contractions, participants made *small* errors (∼2°) in perceived index finger position in the direction of elbow flexion, *regardless* of the contraction type. There was little to no effect of either contraction type on drift in perceived index‐finger position in our test. Our results support the view that high‐level proprioceptive judgements of hand position can be minimally affected by the effects of muscle thixotropy. Thus, we suggest that muscle spindle signals do not dominate the central, cross‐modal transformations of sensory information that are required for high‐level proprioceptive judgements.

## Abstract (highlights)

- **What is the central question of this study?**
  What is the effect of thixotropic conditioning on drift and accuracy on the perception of limb position?
- **What is the main finding and its importance?**
  Thixotropic conditioning of elbow flexor or extensor muscles does not differentially affect perceived forearm position, when judged using a high‐level proprioceptive task. Any systematic effect of thixotropic conditioning on accuracy is small. Further, proprioceptive drift in limb position was not present in the high‐level judgements used. The results provide support for the existence of two (or more) ways that limb position can be perceived and point to the need for more studies to explore the boundary conditions for the observations.

## 1 INTRODUCTION

Despite its fundamental importance, the accuracy of our sense of limb position depends on the prior muscle ‘conditioning’ of the limb (Proske et al., 2014) and hence the exact methods used to test it. Our perceived limb position can be manipulated by changing the thixotropic state of muscles, particularly their spindle endings.

A prior voluntary contraction, a prior muscle length change or a combination of the two alters the passive properties of muscle spindle receptors (Hill, 1968; Proske et al., 1993). If a muscle is passively shortened after an isometric voluntary contraction, the muscle and its spindles become slack, and their discharge rate declines (Burke & Gandevia, 1995). Conversely, if a muscle is passively stretched after an isometric contraction, contraction‐induced bonds will be broken, thus abolishing the effect of the previous muscle contraction (Héroux et al., 2020). Thus, the discharge rate of muscle spindles and their sensitivity to stretch depends on the size and the speed of previous length changes as well as any previous contraction (Matthews, 1972). The discharge rate of muscle spindle is interpreted by the central nervous system (CNS) as a change in joint angle (Clark et al., 1985). Thus, deliberate experimental manipulation of a muscle's history of contraction and stretch, referred to as thixotropic conditioning, can change the accuracy of a person's perception of joint angle (Allen et al., 2007; Gooey et al., 2000).

However, thixotropic conditioning may not influence all judgements of limb position. There are two types of proprioceptive judgements: low‐level judgements and high‐level judgements (Héroux et al., 2022, 2024). In a low‐level judgement of limb position, a person discriminates between or matches proprioceptive signals, for example, reporting an elbow joint angle by flexing or extending at the elbow to align one forearm with the other, unseen forearm. In a high‐level judgement of limb position, a person reports the position of their body relative to their central representation of the external world (Ghafouri et al., 2002; Panday et al., 2014) by integrating proprioceptive signals into their central representation of their body (Longo & Haggard, 2010), for example, reporting an elbow joint angle by aligning a paddle with their unseen forearm (Tsay et al., 2016a). There are differences in the accuracy and variability of low‐ and high‐level proprioceptive judgements of limb and hand position (Héroux et al., 2024; Ingram et al., 2019; Proske & Chen, 2021), and this has been used to indicate that these decisions are made using distinct physiological processes (Héroux et al., 2024).

Importantly, the influence of thixotropic conditioning may differ between two types of proprioceptive judgements of limb position. In low‐level proprioceptive judgements, conditioning contractions create directional errors in accuracy of several degrees (Allen et al., 2007; Gooey et al., 2000; White & Proske, 2009). For example, conditioning the elbow flexor muscles using a concentric contraction induces an error in the direction of extension in a joint position (left–right) *matching* task; conversely, conditioning the elbow extensor muscles induces an error in the direction of flexion. In contrast, in high‐level proprioceptive judgements, conditioning contractions create small unidirectional errors, inconsistent with spindle‐based effects (Tsay et al., 2016a, 2016b).

It has also been suggested that thixotropic conditioning of muscles can induce directional drifts in proprioceptive signals over time (Proske et al., 2014). Although small in amplitude, thixotropy‐related drift has been observed in an upper limb low‐level proprioceptive matching task (Tsay et al., 2014). In contrast, using a measure of the perceived distance between the two index fingers in the horizontal plane, we failed to find drift over a 3 min period using a visual (high‐level) task (Rana et al., 2020).

Here, we searched for an effect of two forms of thixotropic conditioning (elbow flexion and elbow extension) on changes in the perceived position of the finger over time (i.e. drift) and actual perceived position (accuracy) measured using a high‐level proprioception task (visual indication of finger‐tip position). Participants were deliberately never shown the precise position and configuration of their upper limb. Results from these two types of thixotropic conditioning were compared with those from the control condition, in which no muscle contraction or conditioning occurred.

## 2 METHODS

### 2.1 Ethical approval

The study was approved by the University of New South Wales Human Research Ethics Committee (HC190840) and participant consent was obtained in writing prior to the experiment. The study was conducted according to the Declaration of Helsinki (2024), but with omission of clause 35.

### 2.2 Participants

We recruited 25 healthy adult participants (mean age 32.6 years, SD 8.0 years; 11 males, 21 right‐handed). We have previously used a similar sample size to study the perceived upper‐limb position in the horizontal workspace (Qureshi et al., 2018) and obtained precise estimates of perceived index finger location (0.80–1.25 cm margin of error, which represents one‐half of the 95% CI). Participants were excluded if they had any major musculoskeletal or neurological conditions that would impact sensory or motor hand function. Examples of such conditions include a recent wrist or hand fracture, stroke and sensory loss due to diabetes. Participants were offered $20 AUD per hour to participate in the study.

### 2.3 Experimental set‐up

**FIGURE 1:**
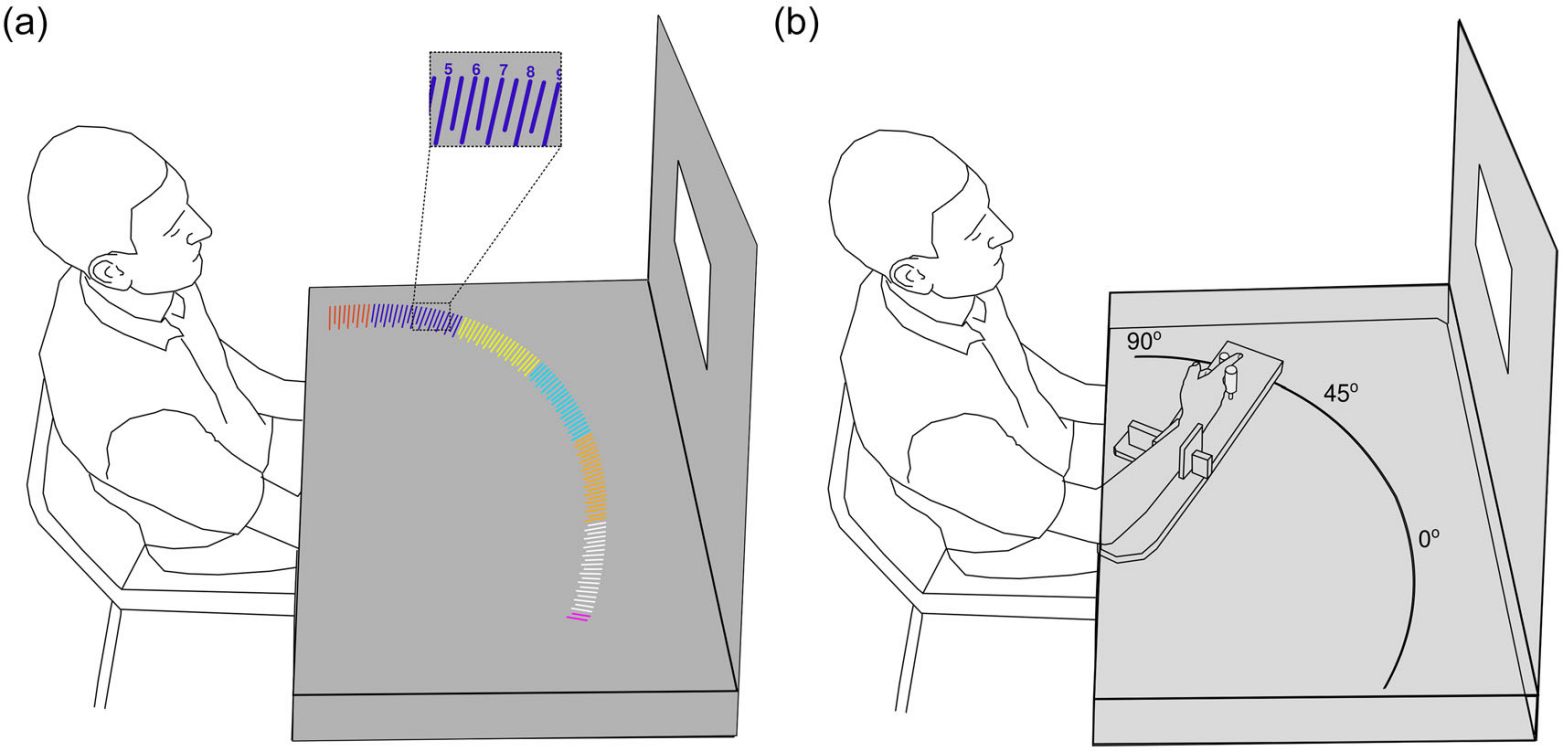
A participant seated in the experimental set‐up. (a) The horizontal surface that occluded participant vision of their arm is rendered grey. The offset rectangle depicts a magnification of projected angles with their associated labels, with which the participant made a judgement of their perceived index finger position. The black fabric that occluded participant vision of their body and the room is not shown. (b) The horizontal surface that occluded participant vision of their arm is rendered transparent grey. The arm positions that represented 0°, 45° and 90° are labelled, but were not visible to the participant. Without the participant's knowledge, their arm was positioned so that their index finger was directly under the line projected at 45° for every judgement.

We used a simple visual matching task in which participants identified the angular location of their index tip. Each participant was seated in front of a table enclosed in a booth of black cloth (Figure 1). On the table was a rotating armrest that could be locked in place at different angles. A horizontal black surface occluded the participant's vision of the armrest. The experimenter used a permanent marker to draw the line of the long axis of the tip of the participant's right index finger and instructed the participant to pay attention to this line, as they would use it to make judgements later in the experiment. Then, the experimenter placed the participant's right forearm in the armrest with the participant's shoulder flexed to about 45°. A black cloth hid the participant's upper‐arm and shoulder from view. The participant's elbow was placed at the axis of rotation of the armrest (flexion/extension), and the participant's hand was placed in a ‘pistol’ position. Supports were placed at the interphalangeal joints of their index finger so that it was held passively extended, and all other finger joints rested in a natural, flexed position. The participant's wrist was supported at the front and back by a padded plate. A projector was placed 1.5 m above the table, and a computer monitor was placed about 1 m in front of the participant at eye‐level. A small camera provided a continuous video stream of the participant's hand, visible only to the experimenter, to ensure it did not move at key times in the experiment.

Electromyographic signals (EMG) were recorded from the muscle belly of the right biceps brachii and triceps brachii muscles with pairs of Ag–AgCl electrodes (diameter 10 mm, spacing 30 mm; Cleartrace; ConMed Corporation, Utica, NY, USA). The ground electrode was placed over the right clavicle. EMG signals were amplified (×1000), band‐pass filtered (20–500 Hz; isolated preamplifier, 1902; CED, Cambridge, UK) and sampled at 2 kHz.

### 2.4 Maximum voluntary contraction

To determine EMG amplitude of maximal biceps and triceps brachii muscle contractions, the participant performed three maximal voluntary elbow flexion contractions, followed by three maximal voluntary elbow extension contractions. They were performed with the participant's arm in the experimental set‐up, with the armrest locked in position at 45° elbow flexion. A 30‐s rest period was provided between contractions. Elbow flexor and extensor EMG were digitally rectified and smoothed (0.1 s time constant), and the maximal EMG amplitude (EMG_max_) across all contractions was determined for each muscle.

Next, 20% of the participant's maximal biceps and triceps brachii muscle EMG amplitude was calculated. Real‐time visual feedback of the digitally rectified and smoothed flexor and extensor EMG signals was displayed on the computer monitor in front of the participant, as was a target line at their 20% EMG_max_. The participant practised 20% EMG_max_ contractions of elbow flexion and extension until they could consistently reach a 20% EMG_max_ contraction in ∼2 s and slowly relax over ∼4 s until their muscle was passive.

### 2.5 Judgements of perceived index finger position

In each judgement, a circle sector spanning 0° to 110° was projected onto the black surface above the participant's upper limb. The centre of the circle was directly above the participant's elbow, and its circumference was marked up with ticks at 1° intervals. The radius of the sector was adjusted so that it was equal to the distance between the participant's elbow and the tip of their index finger. In this way, the ticks acted as a ‘protractor’ that could be used by the participant to indicate the perceived position of their hidden index finger (Figure 1b), and thus the configuration of their upper limb. The ticks were grouped in blocks of different colours, and every second tick in a block was labelled with an integer from 0 to 9. Labelled ticks were 2.2 cm long, whereas unlabelled ticks were 1.6 cm long.

The participant made each judgement by verbalising the colour and number associated with the tick that they perceived was directly above their hidden index finger. To select an unlabelled tick, the participant added 0.5 to the closest labelled tick in an anticlockwise direction. For example, to select the unlabelled tick between green rays labelled 1 and 2, the participant reported ‘green 1.5’. The order of block colours, and the number label of the first tick were randomised in each judgement. After the participant made a judgement, the selected tick was projected onto the black surface and the participant was prompted to confirm their selection. The participant practised judgements of upper‐limb position until they could consistently indicate the perceived position of their index finger in ∼3 s.

### 2.6 Trial procedure

The participant completed five blocks of trials. At the start of each trial, the experimenter asked the participant to close their eyes, placed the participant's arm at a random angle, and then passively moved the participant's elbow back and forth at a rate of ∼20°/s. This movement was either an extend–flex cycle, where the elbow was extended to 0°, flexed to 80° and then returned to the test angle, or a flex–extend cycle, where the elbow was flexed to 80°, extended to 0° and then returned to the test angle. Passive movement cycles (i.e. flex–extend, extend–flex) were randomised in each trial. The purpose of these movements was to standardise the thixotropic state of the elbow flexor and extensor muscles at the start of each trial. EMG was used to ensure the participant's elbow flexor and extensor muscles were relaxed during these movements; if there was muscle activity that was greater than 1% of the participant's EMG_max_ and lasted longer than 2 s in either the flexor or extensor muscles, the passive movements were restarted. There were three types of trial: flexion, extension and control.

#### 2.6.1 Flexion and extension trials

The experimenter asked the participant to close their eyes, and then passively moved the participant's upper limb back and forth as described above. After the passive movements, the experimenter placed the participant's arm at 45° elbow flexion, so that the long axis of their index finger was directly underneath the line that would be projected at 45°. The participant then opened their eyes and performed a conditioning contraction to the level of 20% EMG_max_ with the muscle specified by the trial type, either their elbow flexors or elbow extensors. Once their elbow flexor and extensor muscles were completely relaxed, the experimenter projected the ‘protractor’ lines onto the black surface, and the participant made the initial judgement of perceived index finger position (time‐0). Sixty seconds later, the participant made a second judgement of perceived index finger position (time‐60). In both judgements of perceived index finger position, the long axis of the participant's index finger was directly underneath the line that was projected at 45°. During the 60 s delay between judgements, the participant watched a silent film on the monitor in front of them to standardise their attention.

#### 2.6.2 Control trials

The experimenter asked the participant to close their eyes, and passively moved the participant's upper limb back and forth before placing it at 45° of elbow flexion. Then, the experimenter instructed the participant to open their eyes. After a delay of 6 s, the experimenter projected the ‘protractor’ onto the black surface, and the participant completed an initial judgement of perceived index finger position (time‐0). The 6 s delay was to account for the absence of muscle contraction in control trials, so that the initial judgement (time‐0) occurred at approximately the same time in all trial types. As in the flexion and extension trials, 60 s after their initial judgement of index finger position, the participant completed a second judgement of perceived index finger position (time‐60). Again, as in flexion and extension trials, the long axis of the participant's index finger was directly underneath the line projected at 45° in both judgements of perceived index finger position. During the 60 s delay, the participant again watched a silent film on the monitor in front of them to standardise attention.

If the participant corrected their judgement, the trial was restarted; this ensured that the participant made each judgement at approximately the same time across trials. Additionally, once the ‘protractor’ was projected, if the participant took longer than 5 s to make their decision, the trial was restarted. We used EMG to monitor muscle activity throughout the trial, and a live video stream of the participant's hand and upper limb to monitor unwanted movement. The trial was abandoned during the conditioning contraction if there was substantial co‐contraction of the antagonist muscle that was greater than 10% EMG_max_ of the agonist muscles, or the participant took longer than 3 s to relax their elbow muscles after the contraction. The trial was abandoned during or between the measures of perceived index finger position if there was a single spike of muscle activity greater than 5% EMG_max_ in the flexor or extensor muscles, the participant took longer than 3 s to make a judgement, or the experimenter observed any movement of their hand or upper limb. Each abandoned trial was restarted.

The participant completed 15 trials in five trial blocks. A single block consisted of a flexion trial, an extension trial, and a control trial completed in random order. Between each block of trials, the participant removed their upper limb from the experimental set‐up and performed a bimanual coordination test in full view of their hands. This ensured the participant was not in one posture for an extended period of time or spent the entire experiment with no visual feedback of the upper limbs. Also, by removing and subsequently replacing the participant's upper limb in the experimental apparatus, we reduced the likelihood that they would realise there was only a single test angle. Indeed, no participant reported they were aware a single test angle was used. For each trial, the angle of the tick that the participant selected at time‐0 and time‐60 was recorded. This was the perceived angle for each judgement. EMG data from the elbow flexor and extensor muscles were also recorded.

### 2.7 Data and statistical analyses

Each of the 25 participants performed 15 trials – total 375 trials. Due to equipment error, three trials were removed for one participant and seven trials for another. Six other trials violated the EMG criteria and were also removed before data analysis. Subsequently, a total of 359 trials from the 25 participants were analysed. Of these trials, *n* = 31 (∼1%) were restarted (13 flexion trials, 11 extension trials, 7 control trials; average across participants 1.2 trials (range: 0–4)).

Prior to data analysis, we reviewed all rectified and smoothed EMG recordings and removed trials in which either of the following occurred in the time between judgements at time‐0 and time‐60: a single burst of elbow muscle activity greater than 5% EMG_max_ or a 1‐s window in which elbow muscle activity was greater than 2.5% EMG_max_. This was done to remove any trials in which an unwanted muscle contraction would have affected the thixotropic conditioning of the elbow muscles. Subsequently, we measured peak agonist and antagonist muscle activity for each 20% EMG_max_ conditioning contraction; group means (SD) are reported for flexion and extension conditioning.

**FIGURE 2:**
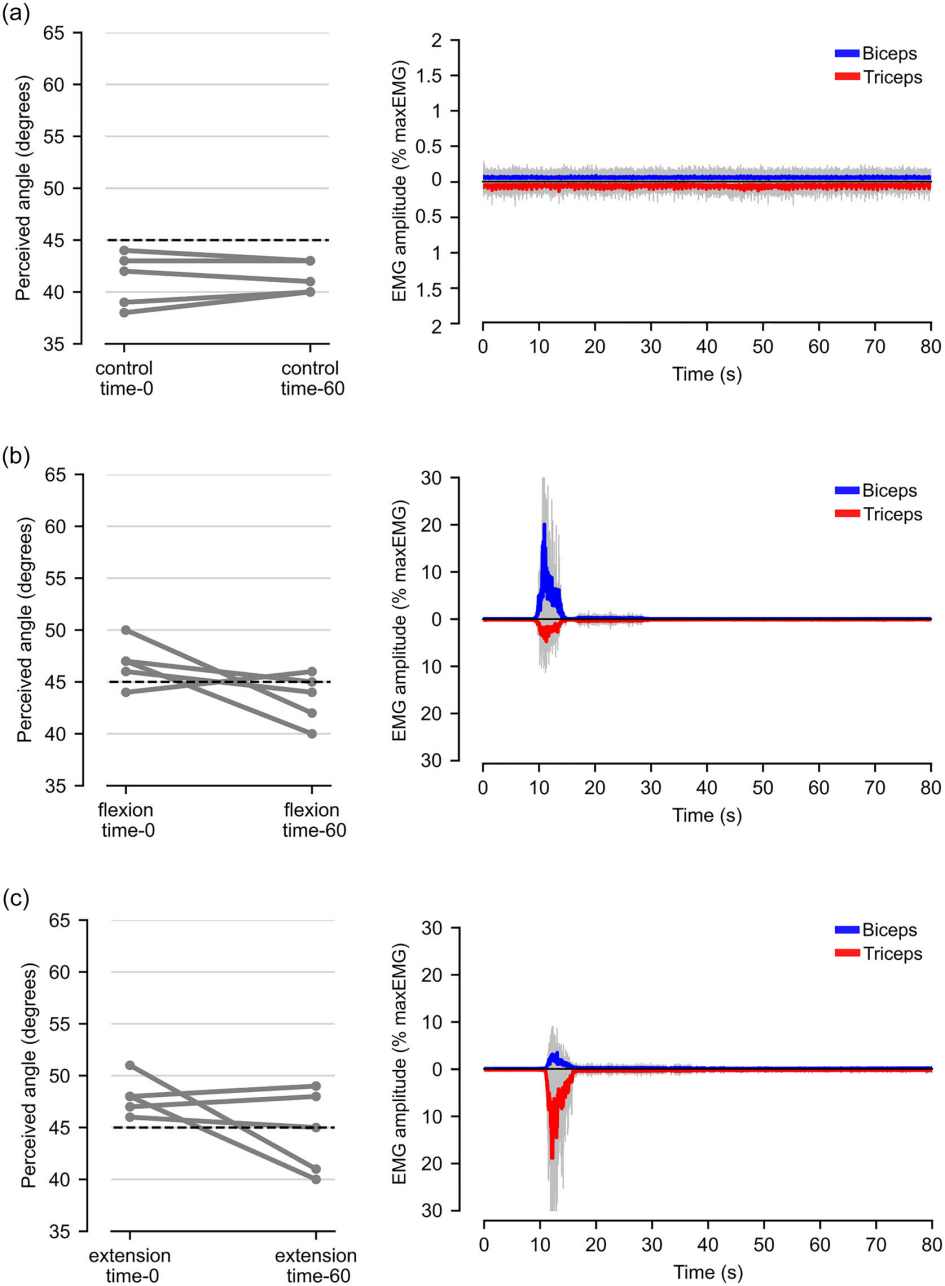
Data from an exemplar participant in (a) control trials, (b) flexion trials, and (c) extension trials. The left column shows the raw data from judgements of upper limb position, with the black dashed line indicating the actual angle of the index finger. Larger angles indicate a more flexed elbow position. The right column shows a rectified EMG trace from a single judgement of upper limb position for each trial type. Coloured traces show EMG data from which the mean has been subtracted, and that have been rectified, smoothed (0.1 s time constant) and expressed as a percentage of the participant's maximum EMG. Grey traces show EMG data from which the mean has been subtracted, and that has been digitally rectified and expressed as a percentage of the participant's maximum EMG. There is a small amount of activity in the antagonist muscle trace for both the flexion and extension trial during the 20% maxEMG contraction.

For each participant, scatter plots of perceived angle data similar to those in the left column of Figure 2 were generated. However, these plots were blinded: they did not specify the experimental condition (i.e. control, flexion, extension) or participant identifier. These scatter plots were inspected by two senior investigators (A.B., M.H.) to identify potential data entry errors or unexpected patterns in participant responses. No data were removed as part of this data analysis step.

To estimate the mean perceived angle selected at time‐0 and at time‐60, we used an intercept‐only linear mixed model for each time point (time‐0 and time‐60) and trial type (control, flexion, and extension), and adjusted for repeated trials within participants.

We calculated the perceived drift in index finger position in each trial as the difference in the perceived angle at time‐60 and time‐0. To estimate the amount of drift in each time point condition, we used a linear mixed model for each trial type (control, flexion, and extension); a random intercept was included to account for the dependence of data within participants.

To estimate the effect of thixotropic conditioning with different contraction types on perceived drift, we used a linear mixed model for each of the following comparisons: flexion trials versus control trials, extension trials versus control trials, and flexion trials versus extension trials. For each comparison, judgements at time‐60 were regressed on judgements at time‐0 and condition, adjusting for repeated trials within participants. Between‐condition mean differences in judgements and 95% confidence intervals are reported. Statistical analysis was performed in Python (v3.12, Python Software Foundation; Python Language Reference; available at http://www.python.org) using the statsmodels package (v0.14.4) for mixed linear models fit by maximum likelihood estimation. Random intercepts were included for participants in all models.

Results are reported as mean differences [95% CI]. Narrow confidence intervals signify more precise estimates. Confidence intervals of mean differences that cross zero indicate (little to) no difference in the means of the outcomes between conditions. Negative angle values indicate an error or drift in the direction of elbow extension, while positive values indicate an error or drift in the direction of elbow flexion.

## 3 RESULTS

Some participants made consistent judgements across trials, while others varied between trials. Perceived angles in each trial of a representative participant in each condition are shown, along with exemplar EMG traces from each contraction type (Figure 2). This illustrates the variation between trials.

**FIGURE 3:**
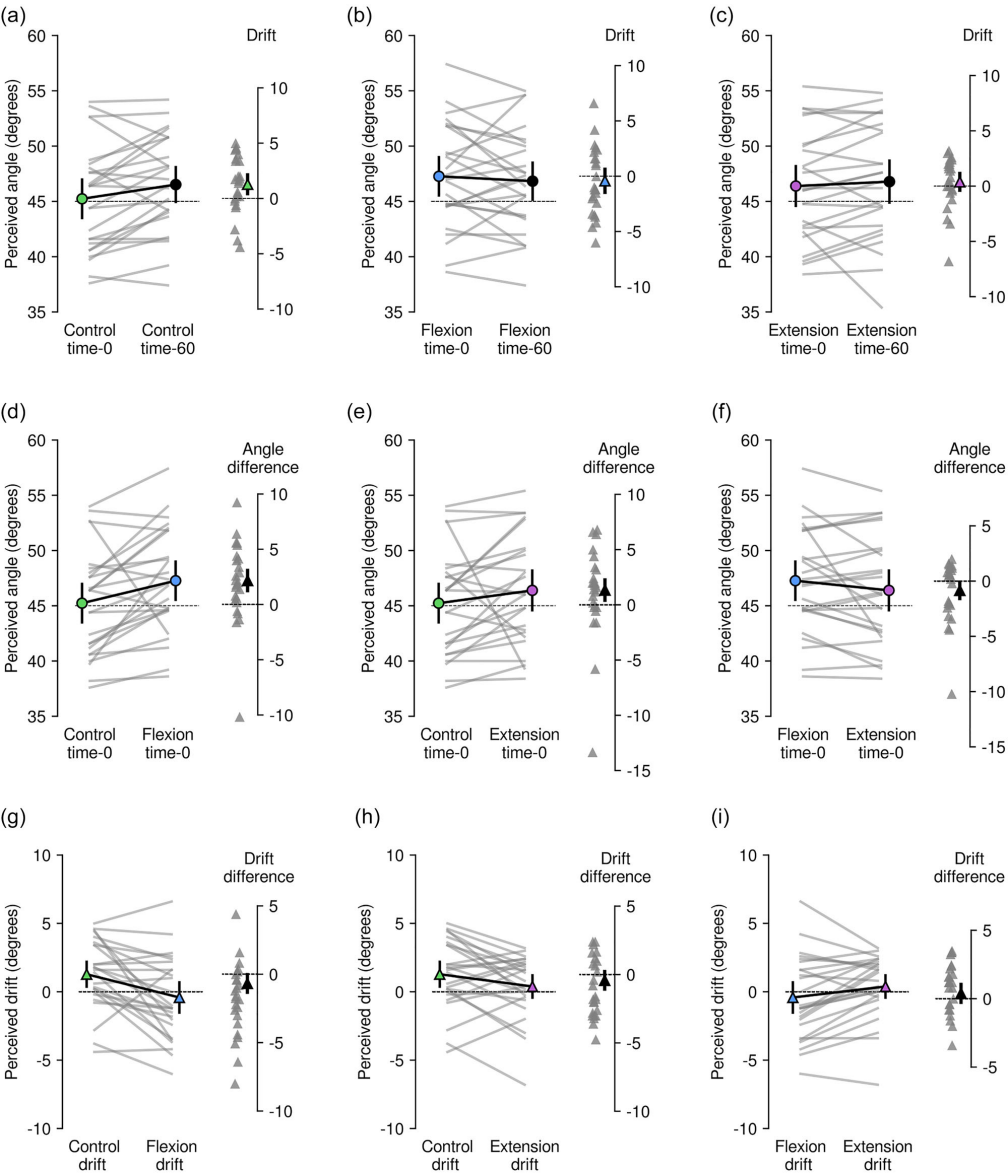
Top row, perceived angles and drift in each trial type: (a) control trials, (b) flexion trials, and (c) extension trials. Middle row, comparison of perceived angle at time‐0 between trial types: (d) control and flexion trials, (e) control and extension trials, and (f) flexion and extension trials. Bottom row, comparison of perceived drift between trial types: (g) control and flexion trials, (h) control and extension trials, and (i) flexion and extension trials. All means are shown with their 95% confidence intervals (black error bars) derived from each linear mixed model. Individual participant mean values are shown by grey lines and single values by grey triangles. Coloured circles show group mean perceived angle at time‐0 in control (green), flexion (blue) and extension trials (purple); black circles show group mean perceived angle at time‐60 in control, flexion and extension trials; coloured triangles show group mean drift in control (green), flexion (blue) and extension trials (purple); black triangles show group mean difference in perceived angles at time‐0 (d, e, f) and group mean difference in drift between contraction types (g, h, i). Black dashed lines show the mean actual angles of participants’ index fingers (left line) and the line of no difference (right line). Negative angle values indicate an error or drift in the direction of elbow extension.

To gain a full picture of the results, Figure 3 shows the mean data for everyone for all conditions at time‐0 and time‐60. It is plotted in three ways: perceived angle and angular drift within a condition (i.e. for the control, flexion and extension condition, Figure 3a, b and c, respectively), perceived angle and the angular difference between conditions (control vs. flexion; control vs. extension, and flexion vs. extension, Figure 3d, e and f, respectively), and finally, the angular drift and the difference in angular drift (for control vs. flexion; control vs. extension, and flexion vs. extension, Figure 3g, h and i, respectively).

The main finding is that in all conditions, errors in perceived index finger position, and changes in perceived index finger position over time, i.e. drift, were small. We assessed drift that might occur over 60 s in the control condition and observed only a small change (Figure 3a). Here, the mean perceived angle at time‐0 was 45.2° [95% CI 43.4° to 47.1°], and 46.5° [44.8° to 48.2°] at time‐60, with a drift towards flexion of 1.3° [0.3° to 2.3°] over 60 s. For the flexion condition (Figure 3b), the mean perceived angle at time‐0 was 47.2° [45.4° to 49.1°], and 46.8° [45.0° to 48.6°] at time‐60, with little to no overall drift (−0.4° [95% CI −1.6° to 0.8°]). For the extension condition (Figure 3c), the mean perceived angle at time‐0 was 46.4° [44.5° to 48.3°], and 46.8° [44.8° to 48.8°] at time‐60, with little to no overall drift (0.4° [−0.5° to 1.3°]).

Simply contracting the elbow flexors before a measure of perceived index finger position seemed to cause participants to perceive their elbow as being slightly more flexed at time‐0, but there was no drift in this misperception over 60 s (Figure 3b, c). This effect was present regardless of whether the comparison was between the flexion and control conditions (2.1° [1.1° to 3.2°]; Figure 3d), or between the extension and control conditions (1.3° [0.3° to 2.4°]; Figure 3e). Furthermore, there was little to no difference in the effect of elbow flexor muscle contraction and elbow extensor muscle contraction on perceived angle at time‐0 (−0.8° [−1.7° to 0.1°] (Figure 3f). This suggests that contraction‐induced changes in muscle spindle discharge of elbow muscles were having minimal effect on perceived finger position.

We observed little to no apparent effect of elbow muscle conditioning on drift of the perceived index finger location. When the drift in the control condition was subtracted from the drift in the flexion condition (Figure 3g), the mean difference in drift was −0.7° [95% CI −1.5° to 0.1°]. When the drift in the control condition was subtracted from the drift in the extension condition (Figure 3h), the mean difference in drift was −0.4° [−1.2° to 0.3°]. Finally, when the drift in the flexion condition was subtracted from the drift in the extension condition (Figure 3i), the mean difference in drift was 0.4° [−0.4° to 1.2°]. Thus, whether assessed within a condition or between conditions, there was no evidence for consistent drift over 60 s.

## 4 DISCUSSION

This is a descriptive study of the effect of thixotropic conditioning of the elbow muscles on the accuracy of judgements of index finger position and an assessment of whether the perceptual judgements drifted over a minute. The judgements of position were made by reporting with a visual frame of reference, and hence in the terminology of Héroux et al. (2022) they are of a higher level than say a simple left‐to‐right match of position with no involvement of vision.

Based, on the precision of our results (see Figure 3), we conclude that, compared to no prior contraction of the elbow muscles, there was only a small bias in perceived position with a prior elbow extension or flexion contraction (∼2–3°). This was a bias into flexion. A differential effect of flexion versus extension conditioning would have been expected for judgements of arm position based on simple left–right matching (Héroux et al., 2022; Proske & Chen, 2021; Roach et al., 2023). There have been other reports of similar small effects of conditioning contraction of upper limb muscles with the direction of bias showing no difference between flexion and extension conditioning (e.g. Roach et al., 2023).

Our results also reveal the variation between participants in the perceived position of the hand. The reference position was set at 45° elbow flexion – yet individuals varied over ∼20° in their mean selected perceived position. Not unexpectedly, the mean for the group was close to a veridical judgement. Such large variation between participants has been documented for other proprioceptive judgements in the upper limb (e.g. thumb–index distance for a grasp) (Butler et al., 2019; Héroux et al., 2024) (see also Jastrow, 1886). This spread of judgements between individuals typifies those made when there is some form of cross modal interaction (e.g. between visual and proprioceptive frames of reference).

Second, the precision of our results also allows us to conclude that while in the control task there was a small degree of drift over 60 s (∼1–2°), such drift did not occur with either flexion or extension conditioning and, unsurprisingly, there was no difference between the two forms of conditioning. The lack of drift in this task is like the result of Rana et al. (2020) who found that the perceived horizontal distance between the two hands drifted minimally over 3 min, as did the perceived position of one finger.

With regard to thixotropic conditioning, our study adds to the debate about the circumstances under which thixotropic conditioning produces the changes in position sense that are predicted by its alteration of the firing of muscle spindle afferents. Thixotropic conditioning has frequently been shown to alter perceived position in left‐to‐right position matching tasks (Allen et al., 2007; Proske & Chen, 2021; Tsay et al., 2016a, 2016b) and, in one study, of pointing to indicate joint position (Walsh et al., 2009). However, there are a growing number of studies in which a conditioning effect is absent. These have assessed position sense at the elbow, usually with the upper limb acting against gravity, and when the judgements are made by pointing with the other (unconditioned) arm (Proske & Chen, 2021; Roach et al., 2023; Tsay et al., 2016a). As a result, Proske and colleagues have argued that the thixotropic effect attributable to muscle spindles can be over‐ridden when judgements involve a visual component such as pointing with the other arm or verbally describing a visual judgement (Proske & Chen, 2021; Proske & Gandevia, 2018). Hence, our study provides a new example of this likely over‐riding of muscle spindle contributions to position sense. Such situations presumably require central interactions involving both proprioceptive signals and signals related to other frames of reference (e.g. vision) (Buneo & Andersen, 2012; Vallar, 1997).

With regard to proprioceptive drift, our study provides a critical observation on the absence of a drift in a high‐level judgement of hand position with or without any form of muscle conditioning. In a simple left‐to‐right matching task of elbow position when the thixotropic conditioning of the flexor or extensor muscles was carefully controlled, only a small drift attributable to a spindle receptor mechanism has been observed over 5–10 s (Tsay et al., 2014). As indicated above, absence of drift in the present task fits with observations on the lack of drift for a high‐level judgement of finger position within the usual workspace of the hands. In both, sustained vision of the body part was deliberately prevented. However, the literature on proprioceptive drift is mixed, with drift and changes in accuracy of judgements changing over time being reported in some but not other studies (for details see Paillard & Brouchon, 1968; Velay et al., 1989). The explanation for the discrepancy has not yet been revealed, but it may depend on an interaction between prolonged vision and memory of limb position.

Some methodological factors are notable in our research design. First, we used strict criteria based on the level of EMG in the elbow flexors and extensors to ensure that the muscle spindles were in a consistent state for the three experimental conditions. This should have acted to reduce suprathreshold ‘noise’ in the decision‐making process (Faisal et al., 2008; Stein et al., 2005). Muscle activity recordings enabled us to standardise the intensity of the conditioning contractions and to confirm that elbow flexors and extensors were relaxed following each contraction. In this study, we selected a conditioning contraction at 20% EMG_max_ because: (1) this intensity induces thixotropic effects equivalent to those elicited by 50% maximal contractions (Héroux et al., 2020); and (2) it reduces the likelihood of antagonist co‐contraction (Pincivero et al., 2019). During practice trials, despite repeated instructions to isolate activation to the target muscle group (i.e. elbow flexors or extensors), EMG activity was frequently observed in the antagonist muscle. While this activity may partially reflect true co‐contraction, it is likely that some was due to cross‐talk (Germer et al., 2021). Could low‐level antagonist activity have influenced our findings? We think this is unlikely. Prior studies have demonstrated robust thixotropic effects on perceived joint angle using 50% maximal contractions (Allen et al., 2007; Gooey et al., 2000; Tsay et al., 2014), a condition under which antagonist activation is more likely (Pincivero et al., 2019). Accordingly, we are confident that the thixotropic conditioning applied in our study was at least as effective as that used in these earlier investigations. Second, the observations were carried out across the horizontal plane so that gravitational torques that required balancing by muscle contraction were eliminated. Third, the readout of the perceived position of the arm involved a randomised protractor that could not be predicted or learned by the participant, and the smallest change that could be selected was 0.5°. This should have contributed to the precision of the measurements. Fourth, no vision of the arm and hand was permitted throughout any trial. Finally, the studies were conducted in a dim room and the participants could not see the main experimental apparatus. Further studies will be needed to determine the relevance of the exact methods used to address the apparent existence of different forms of position sense and some of the discrepancies in the current literature.

In summary, when the index finger is positioned midway through the angular range allowed by elbow flexion (45°), thixotropic conditioning of the elbow flexor or extensor muscles does not differentially affect perceived finger position, when this position is judged using a high‐level proprioceptive task. Any systematic effect of thixotropic conditioning on accuracy is small. Further, proprioceptive drift in the limb position estimates is not a feature in the high‐level judgements we used. Our results provide some support for the existence of two (or more) ways in which limb position can be perceived and point to the need for more studies to explore the boundary conditions for our observations.

## AUTHOR CONTRIBUTIONS

Annie A. Butler, Martin E. Héroux, and Simon C. Gandevia: conception or design of the work. Georgia Fisher, Joanna Diong, Annie A. Butler, Martin E. Héroux and Simon C. Gandevia: acquisition, analysis, or interpretation of data. All authors were involved in drafting the manuscript. All authors have read and approved the final version of this manuscript and agree to be accountable for all aspects of the work in ensuring that questions related to the accuracy or integrity of any part of the work are appropriately investigated and resolved. All persons designated as authors qualify for authorship, and all those who qualify for authorship are listed.

## CONFLICT OF INTEREST

None declared.

## Data Availability

Data are available from the corresponding author.
